# Disease Containment Strategies based on Mobility and Information Dissemination

**DOI:** 10.1038/srep10650

**Published:** 2015-06-02

**Authors:** A. Lima, M. De Domenico, V. Pejovic, M. Musolesi

**Affiliations:** 1 School of Computer Science, University of Birmingham, United Kingdom; 2 Universitat Rovira i Virgili, Tarragona, Spain; 3 Faculty of Computer and Information Science, University of Ljubljana, Slovenia

## Abstract

Human mobility and social structure are at the basis of disease spreading. Disease containment strategies are usually devised from coarse-grained assumptions about human mobility. Cellular networks data, however, provides finer-grained information, not only about how people move, but also about how they communicate. In this paper we analyze the behavior of a large number of individuals in Ivory Coast using cellular network data. We model mobility and communication between individuals by means of an interconnected multiplex structure where each node represents the population in a geographic area (i.e., a *sous-préfecture*, a third-level administrative region). We present a model that describes how diseases circulate around the country as people move between regions. We extend the model with a concurrent process of relevant information spreading. This process corresponds to people disseminating disease prevention information, e.g., hygiene practices, vaccination campaign notices and other, within their social network. Thus, this process interferes with the epidemic. We then evaluate how restricting the mobility or using preventive information spreading process affects the epidemic. We find that restricting mobility does not delay the occurrence of an endemic state and that an information campaign might be an effective countermeasure.

Health and well-being of populations are heavily influenced by their behavior. The impact of the habits and local customs, including patterns of interactions and mobility at urban and regional scales, on health issues is remarkable^1^. The diffusion of mobile technology we are experiencing nowadays gives scholars an unprecedented opportunity to study massive data that describe human behavior^2^. Mobile phones stand out as very personalized devices. Through mobile network data records, phones reveal both the users’ mobility and social network. Data coming from a large number of people can describe trends in the macroscopic behavior of populations^3^,^4^,^5^. The analysis of these trends can be beneficial in a number of real-world scenarios, particularly in domains where cultural and local differences play a central role. It can also provide invaluable support to decision-making, especially in critical situations. For this reason, many public and private organizations are increasingly adopting a data-centric approach in their decisional process^6^. We believe that this strategy can be particularly useful in developing countries, which might lack the type of infrastructure available elsewhere.

Indeed, among the issues that developing countries are facing today, healthcare is probably the most urgent^7^. The effectiveness of campaigns is often reduced due to low availability or poor quality of statistical data, inherent limits in the infrastructure and difficult communication with the citizens, who might live in vast and remote rural areas. As a result, action plans are difficult to deliver. However, we believe that a data-centric approach can be an innovative and effective way to address these issues.

In this paper, we focus on the containment of epidemics. We use movement data extracted from the registration patterns in a cellular network to evaluate the influence of human mobility on the spreading of diseases in a geographic area. In particular, we utilize this model to investigate how infectious agents might spread to distant locations because of human movement in order to identify optimal strategies that can be adopted to contrast the epidemics. We also evaluate how the collaborative effort of the population can be crucial in emergency scenarios. In countries that are facing development challenges, vaccination campaigns are often hard to advertise to the population. Lack of communication and information is believed to be among the main causes of failure for immunization campaigns. The same applies to awareness campaigns that try to promote prophylaxis procedures that reduce the occurrence of contagion. However, in these cases, we argue that a collaborative effort leveraging individual social ties can be effective in propagating this kind of “immunizing information” to a widespread audience. Moreover, information received by people who are socially close can have a higher chance of leading to an actual action.

There has been an increasing interest on the role that human movement plays in spreading infections within large geographic areas^8^,^9^,^10^,^11^,^12^,^13^,^14^,^15^,^16^,^17^, and also in the impact of human behavior on the spreading itself^15^,^18^. The contributions of our work, with respect to the state of the art, can be summarized as follows.

- We consider a network of geographic metapopulations and we propose an epidemic model which describes how people move between different geographic regions and spread the disease. Then, we evaluate containment techniques based on the restriction of mobility in the most central areas. The centrality of the areas is extracted by building a movement network between all the geographic areas based on the mobility patterns of the individuals.
- We extend the model by considering the competing process of information spreading. This process is very different from disease spreading as, unlike disease infection, it does not require user co-location. Rather, information can be transmitted at distance, through a telephone network. Therefore, we investigate *distance contagion* that happens in the communication/information layer. To summarize, we study the dynamics of the system considering three characterizing aspects of the problem: the disease epidemics, human mobility and information spreading. The latter represents the diffusion of information concerning the preventive and curative measures, such as information about the ongoing vaccination and prevention campaigns in a certain area or actions that will help to limit the spread of the infection.
- We construct a multiplex network^19^,^20^,^21^,^22^,^23^, where nodes exist in different networks simultaneously, to model the coupling and the mutual interactions between mobility and communication layers.
- We evaluate our models using the “2013 Data for Development (D4D)”^24^ challenge dataset, provided by Orange, one of the world-leading telecommunication providers. We discuss the effectiveness of the containment strategies and, in particular, for the information dissemination strategy, we evaluate to which extent it contains the disease spreading. We observe that restricting mobility by disallowing any movement from and to a limited set of sub-prefectures does not delay the occurrence of the endemic state in the rest of the country. We also find that a collaborative effort of prevention information spreading can be a more effective countermeasure.

This work is an extension of the original unpublished submission^25^ to the Data for Development Challenge organized by Orange^24^. Finally, it is worth noting that after the challenge a similar approach to the modeling of epidemic spreading and awareness information has been presented in^26^.

## Results

**Figure 1:**
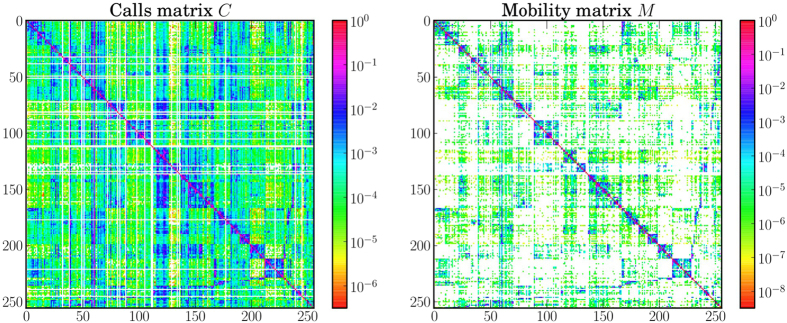
Logarithmic representation of the mobility matrix (**a**) and the call matrix (**b**). Null values are indicated using the white color. For both matrices highest values are mostly concentrated along the diagonal, showing that communication and movement between sub-prefectures is highly uncommon. However, the calls matrix is visibly denser than the mobility matrix, confirming that phone contacts between different sub-prefectures are more usual than movement.

**Figure 2:**
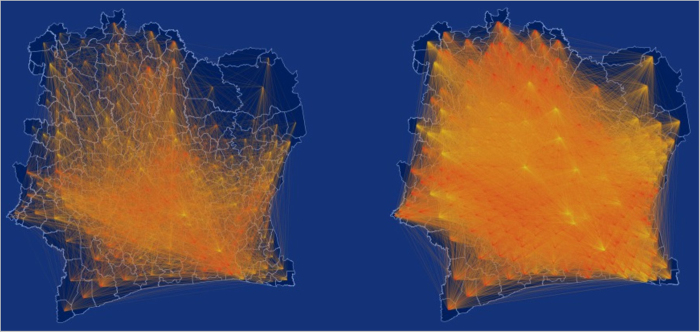
Geographic network obtained from mobility traces (**a**) and call logs (**b**), where nodes represent sub-prefectures. This map was generated by a custom d3 script. Map data: © OpenStreetMap contributors, available under the Open Database License.

A call detail record (CDR) contains information about each phone call, and includes, among other fields, calling and called party phone numbers, time and duration of the calls, and a coarse-grain location of the communicating parties. We extract patterns of individual mobility and communication from the CDRs (our methodology is described in detail in the *Methods* section), and obtain two matrices, the *mobility matrix* 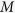 and the *communication matrix* 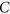, shown in Fig. 1. They represent, respectively, the mobility flow and the communication flow between geographic areas. In Fig. 2 we show the geographic networks of mobility (left panel) and calls (right panel), respectively, where nodes are positioned using the geographic locations of the sub-prefecture they represent.

In the following we will present two models: a model of disease spreading as a function of the mobility patterns of individuals and a model for information spreading among the same population, considering the underlying social structure. The multiplex structure is used to model the interactions between the mobility layer, which represents movement of users between regions, and the communication layer, which captures calls between regions and, consequently, information spreading across the country. Each model will be evaluated using the data inferred from the CDRs, described in the Methods section.

### Epidemic Spreading and Mobility Model

We now present a model that represents the evolution of an epidemic taking place on a network of metapopulations. The aim of the model is to describe how the system evolves under the action of two processes, contagion and mobility. The population is distributed in *n* different metapopulations, where *i*-th metapopulation has *N*_*i*_[*t*] individuals at time *t*. We make the simplifying assumption that there are no deaths and births in the considered time window, i.e., at each time *t* = 1,2,…*T* the total population is constant 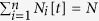.

We assume that contagion happens inside each metapopulation following a standard SIS model^27^. We indicate the number of infected and susceptible individuals at time *t* in a metapopulation *i* with *I*_*i*_[*t*] and *S*_*i*_[*t*], respectively. At each time *t* a person is either infected or susceptible, therefore *N*_*i*_[*t*]=*I*_*i*_[*t*]+*S*_*i*_[*t*].

Simultaneously, individuals move through the metapopulation network according to the *mobility matrix M* of dimension *n*×*n*. The generic element *m*_*ij*_ of the matrix represents the probability that a person moves from the metapopulation *i* to *j*. Hence, the state variables *N*_*i*_[*t*] evolve over time as follows: 
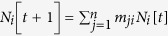
. Under the assumption that individuals inside the classes *I* and *S* move consistently, we can write the last relation also for the state variables *I*_*i*_[*t*] and *S*_*i*_[*t*]. This assumption can be relaxed if data about the behavior of different classes of individuals is available, i.e., when a different matrix *M* can be defined for each class – 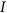 and 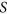. The contagion-mobility combined system can be described by the following set of equations:
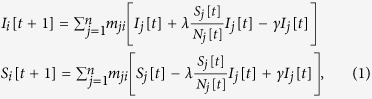
for each metapopulation *i* = 1,2,…,*n*, with *λ* being the product of the contact rate and the contagion probability and *γ* being the recovery rate. The formulae inside the square brackets describe the evolution of *n* SIS models, one for each metapopulation. They are multiplied for the elements of the mobility matrix, which accounts for individuals moving between metapopulations.

This analytical model describes the expected outcome of a stochastic model where the following actions occur at each time step: first, each infected person in the metapopulation *j* causes the infection of new 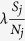 individuals inside *j* (this step is repeated for each metapopulation); successively, a new position *i* is assigned to each individual in the metapopulation *j* according to the probability density function [*m*_*j*1_,*m*_*j*2_,…,*m*_*jn*_] (this step is repeated for each metapopulation).

### Information Spreading Model

The model we presented in the last section describes the spreading of a disease in a population where individuals change locations over time. The aim of this work is to analyze some scenarios and study the effectiveness of possible containment techniques. In particular, as anticipated, we would like to investigate if a collaborative effort of the population is able, in theory, to reduce the spread of the disease and to which extent. The population can disseminate, using pre-existing personal social ties, *immunizing* information (e.g., information about prevention techniques, hygiene practices, advertisement of nearby vaccination campaigns, and in general any information that can lead to a reduction of the number of contagion events).

In order to take into consideration these aspects, we now use a SIR model for each metapopulation in the mobility layer of the multiplex network, so that each person either belongs to the susceptible (S), infected (I) or resistant (R) category. At the same time, another simultaneous epidemic happens on the network of metapopulations, disseminating information that can make individuals resistant to the disease, actually working against the disease epidemics. We model this network by means of a communication layer, where nodes are the same metapopulations existing in the mobility layer.

In fact, a person also belongs to the category of unaware (U) or aware (A) individuals, with respect to the immunizing information. More formally, we have that *N*_*i*_[*t*]=*I*_*i*_[*t*]+*S*_*i*_[*t*]+*R*_*i*_[*t*]=*A*_*i*_[*t*]+*U*_*i*_[*t*].

**Figure 3:**
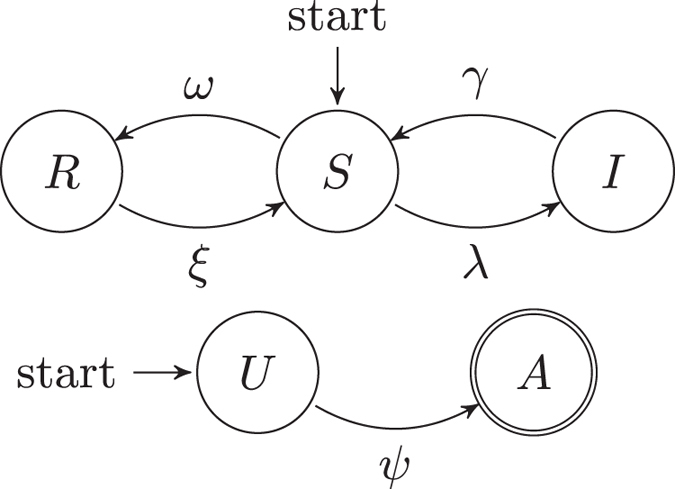
State machines describing the state transitions of a person with respect to the disease contagion (R = Resistant, S = Susceptible and I = Infected) and with respect to the information spreading (U = unaware, A = aware), respectively. A person starts in the susceptible and unaware states. We assume that aware individuals spread the information and cannot go back to the unaware state.

It is worth noting that this “immunizing epidemic” goes *beyond* the boundaries of metapopulations: in other words, it is a *distance contagion*. It is also important to remark that the states “aware” and “resistant” are substantially different. An unaware person that receives the information (i.e., has an “information contact”) becomes aware with rate *ψ*; since the person is aware, he or she will start spreading the information as well. An infected person that receives the information becomes immune with rate *ω*. Additionally, individuals who have acquired immunity through information can lose it with rate *ξ* (for example, because they forget or abandon disease prevention practices). The transition rates between states are summarized in Fig. 3. The model can be described by the following set of equations, specifying how state vectors evolve over time:
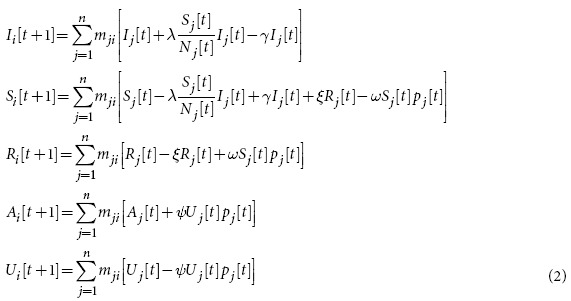
for every *i* = 1,2,…,*n*, where
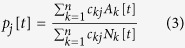
represents the probability that a call from an aware person occurs in the metapopulation *j*. This term is responsible for the interaction between the two layers of the multiplex and it models the distance-contagion. It is possible to verify that if the matrix is identical (absence of contacts between populations) it reduces to *A*_*k*_[*t*]/*N*_*k*_[*t*], falling back to a model where contagion occurs only inside metapopulations.

This analytical model describes the expected value of a stochastic model where the following actions occur at each time step *t*: i) each infected person in the metapopulation *j* causes 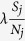 new individuals to get infected inside *j*; ii) each unaware person in the metapopulation *j* becomes aware with probability *ψp*_*j*_[*t*]; iii) each person in the metapopulation *j* who is susceptible, becomes resistant with probability *ωp*_*j*_[*t*]; iv) a new position *i* is assigned to each person in the metapopulation *j* according to the probability density function [*m*_*j*1_,*m*_*j*2_,…,*m*_*jn*_]. It is worth mentioning that each step is repeated for each metapopulation.

### Simulations

The estimated population size of Ivory Coast for July 2012 was 21,952,093^28^. Ivory Coast is organized in 393 sub-prefectures (*sous-prefectures*), which will be used to define metapopulations. Hence, we initialize each scenario by allocating 22 million individuals to sub-prefectures, according to information found in CDRs. In each scenario we bootstrap the spreading process by infecting a fraction of the population (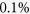) distributed across metapopulations according to different criteria:

- Uniform distribution: every sub-prefecture gets a number of infected proportional to their population, i.e., every sub-prefecture has the same fraction of infected population.
- Random: a single sub-prefecture, chosen randomly, is the origin of the infection.
- Centrality based: the sub-prefectures are chosen according to their centrality rank, in particular focusing on the the first 1, 5 or 10 highest ranked. Although we use eigenvector centrality, we noticed the top ranked sub-prefectures are very similar for other types of centralities, as reported in the supplementary information.

We study the evolution of the epidemics for a period of six months. We investigate multiple scenarios using the analytical model considering a large set of ranges for the values of the key parameters. We conducted a series of Monte-Carlo simulations for multiple sets of parameters, confirming the validity of the analytical models presented in the previous section. In the following, we present results based on these scenarios.

#### Unconstrained Epidemic Spread

**Figure 4:**
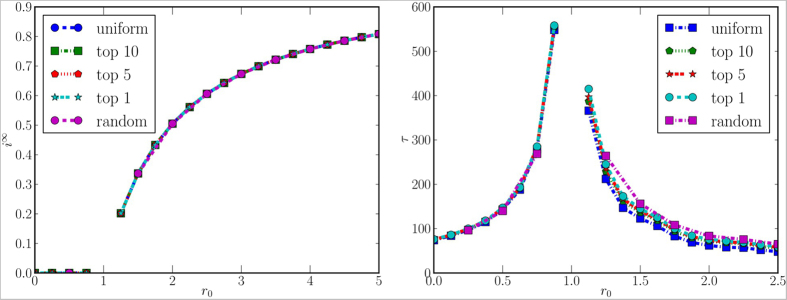
Fraction of infected population at the stationary state (left panel) and time required to reach the stationary state (right panel), for different values of *r*_0_ and for different initial conditions. Missing values in the curves mean that, for the corresponding values, no stationary state is reached during the period of observation.

We will first explore the evolution of the epidemics in the case where no countermeasures are taken. In order to analyze the evolution of the system in more detail, we investigate two metrics: the fraction of infected population *i*^∞^ at the stationary state and the time required to reach the stationary state τ. In Fig. 4 we plot their values versus 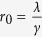, which is the basic reproductive ratio of a classic SIS model^27^. As a future work, we plan to derive the analytical form of the basic reproductive ratio of our models, which take into account mobility and information spreading in the multiplex framework. We observe that for 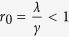 there is no endemic state (i.e., the final fraction of infected population is zero), whereas for *r*_0_ > 1 a non-null fraction of population is infected. Values for *r*_0_ = 1 are missing since no stationary state is reached within our observation window. In other words, for this particular scenario, experimental results show that the basic reproductive ratio of our model is very close to *r*_0_; we expect this to be a consequence of the low inter-subprefectures mobility. We can also notice that the initial conditions do not affect *i*^∞^ at all. Before the critical point (i.e., *r*_0_ = 1) the choice of the initial conditions has also no impact on the delay time, whereas for *r*_0_ > 1 it slightly affects the delay: epidemics that initially involve more sub-prefectures are slightly faster than the others.

#### Geographic Quarantine

**Figure 5:**
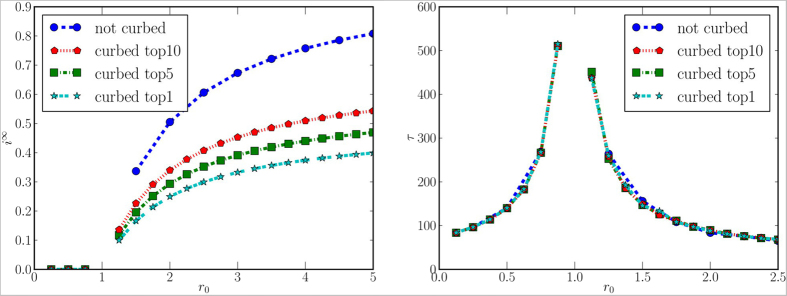
Fraction of infected population at the stationary state (left panel) and time required to reach the stationary state (right panel), for different values of *r*_0_ when the epidemic starts from a random sub-prefecture, and different levels of geographic quarantine are applied. Missing values in the curves mean that, for the corresponding values, no stationary state is reached during the period of observation.

We now analyze the effects of curbing on the mobility between sub-prefectures, i.e., forbidding all the incoming and outgoing movement for a group of sub-prefectures. To this aim, we calculate the centrality values of each sub-prefecture in the mobility network. We present the results for eigenvalues centrality, because the ranking based on other centralities is very similar (see supplementary information). Then, for the quarantine operations, we select those with the highest centrality values. From a practical point of view, this is achieved by simply changing the *i*–th row and column in the mobility matrix, so that all the elements *m*_*ij*_ and *m*_*ji*_ are null, except for the elements *m*_*ii*_ = 1. It is important to note that in this process we recompute *m*_*ji*_s in order to conserve the probability, i.e. so that each row of M sums up to one. For these scenarios, we randomly choose a single sub-prefecture where the initial individuals are infected, and then we average *i*^∞^ and *τ* over all the Monte Carlo simulations. As shown in Fig. 5, the fraction of the infected population is sensibly affected by this measure, as the population inside the quarantined areas is protected from contagion. However, contrary to the intuition, the delay is not affected by the quarantine, even when the countermeasures involve 10 sub-prefectures, which account for almost half population. This suggests that such an invasive, expensive and hard to enforce measure reduces considerably the endemic size, but does not slow down the disease spreading in the rest of the country. For this reason, we now investigate a radically different approach to protect the population.

#### Information Campaign (Social Immunization)

We now show how a collaborative information campaign could help in contrasting the spread of the disease, following the model we presented in the last section. We initialize the scenario by distributing the immunizing information to 1% of the population, randomly chosen regardless of their location. These people will be informed and will be instructed to spread the information. In other words, we assume that they will contact their social connections, according to the call network.

**Figure 6:**
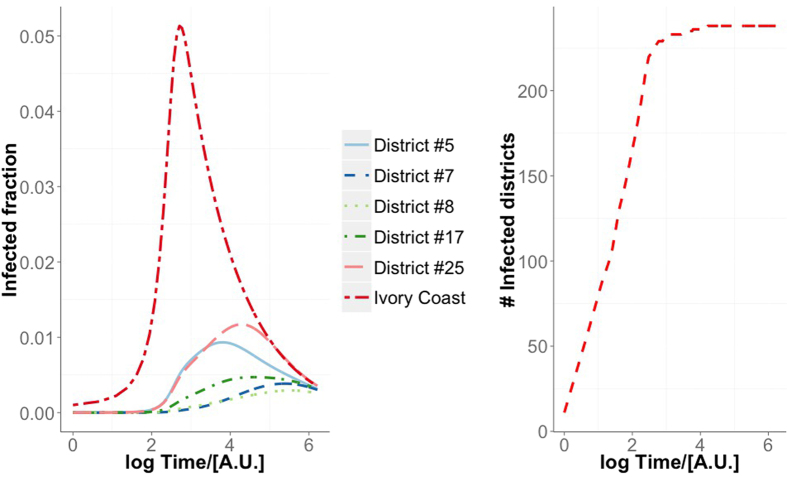
Spatiotemporal evolution of an epidemics starting form the top ten districts ranked by betweenness centrality (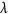 = 1, *γ* = 0.5, *ω* = 0.5, *ψ* = 0.5, *x* = 0.5). The fraction of infected individuals is shown for some districts and the whole Ivory Coast *versus* time (left-hand panel), whereas the number of districts with at least one infected individual is shown on the right-hand panel.

**Figure 7:**
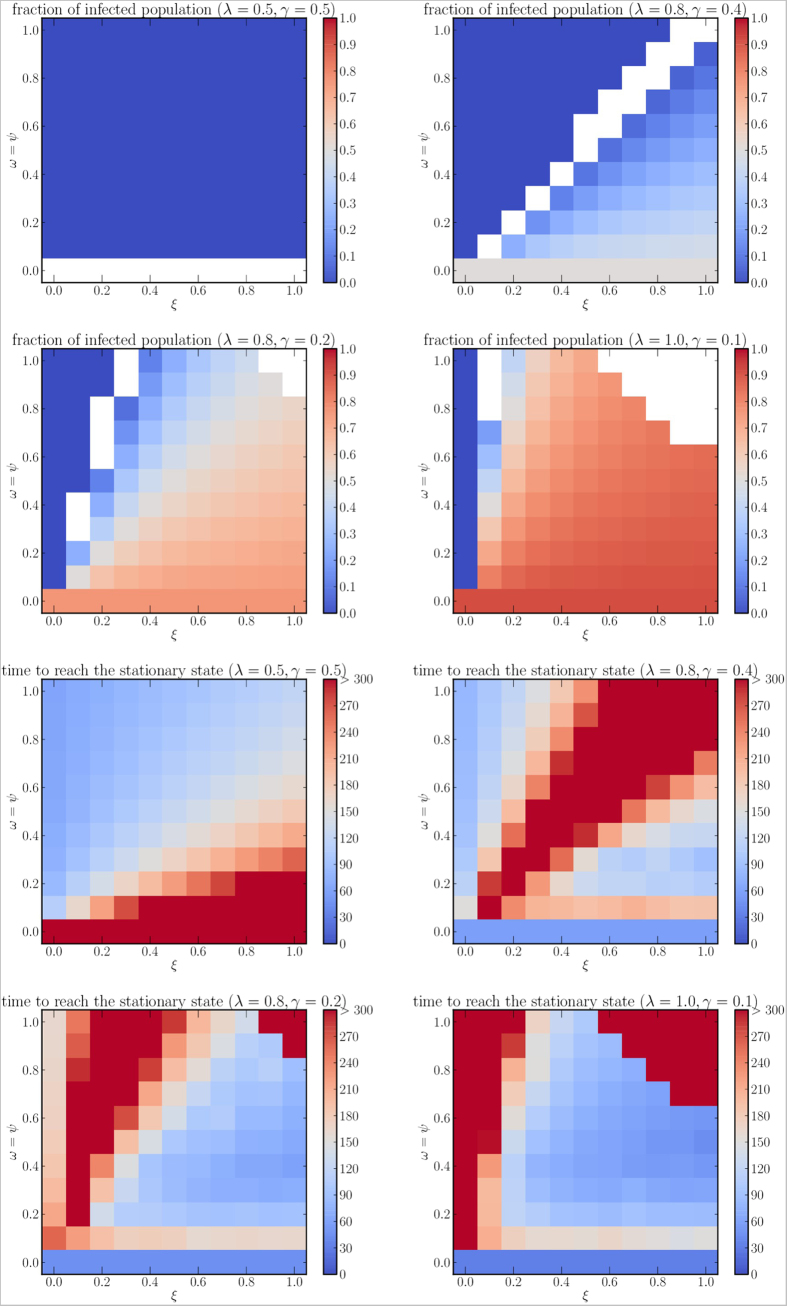
Fraction of infected population at the stationary state (first two rows) and time required to reach the stationary state (last two rows), for different values of 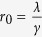 (1, 2, 4, 10, respectively). White spaces show that no stationary state is reached during the period of observation.

In Fig. 6 we present an example of the spatiotemporal evolution of an epidemics for the information campaign case. In Fig. 7 we show the density plots describing *i*^∞^ and *τ* for various values of *r*_0_, for a subset of scenarios where *ω* = *ψ*, i.e., when the information that spreads among the population has the same chance to immunize a person and to involve the person in the spreading process. This is consistent with a scenario where the same set of people who become aware also become immunized by the information they have received. Blank squares show that a stationary state was not reached for the corresponding set of parameters. The figure shows how contagious (*ω* = *ψ*) the immunizing information has to be with respect to how often people “forget” (*ξ*) in order to slow down the disease considerably and to reduce the endemic cases. When *ω* = *ψ* = 0 we fall back to the model without information spreading, and the value of *ξ* does not affect *i*^∞^ and *τ*. For *ω* = *ψ* *>* 0 and *ξ* = 0 the fraction of infected population goes to zero in all cases, because the number of people aware of the information does not decrease, thus increasing the number of new immunized individuals at each step. We can notice that even for low values of participation *ω* and for information that gives temporary immunization (*ψ* > 0), the final fraction of infected individuals is considerably lower than in the case where no countermeasures are taken.

**Figure 8:**
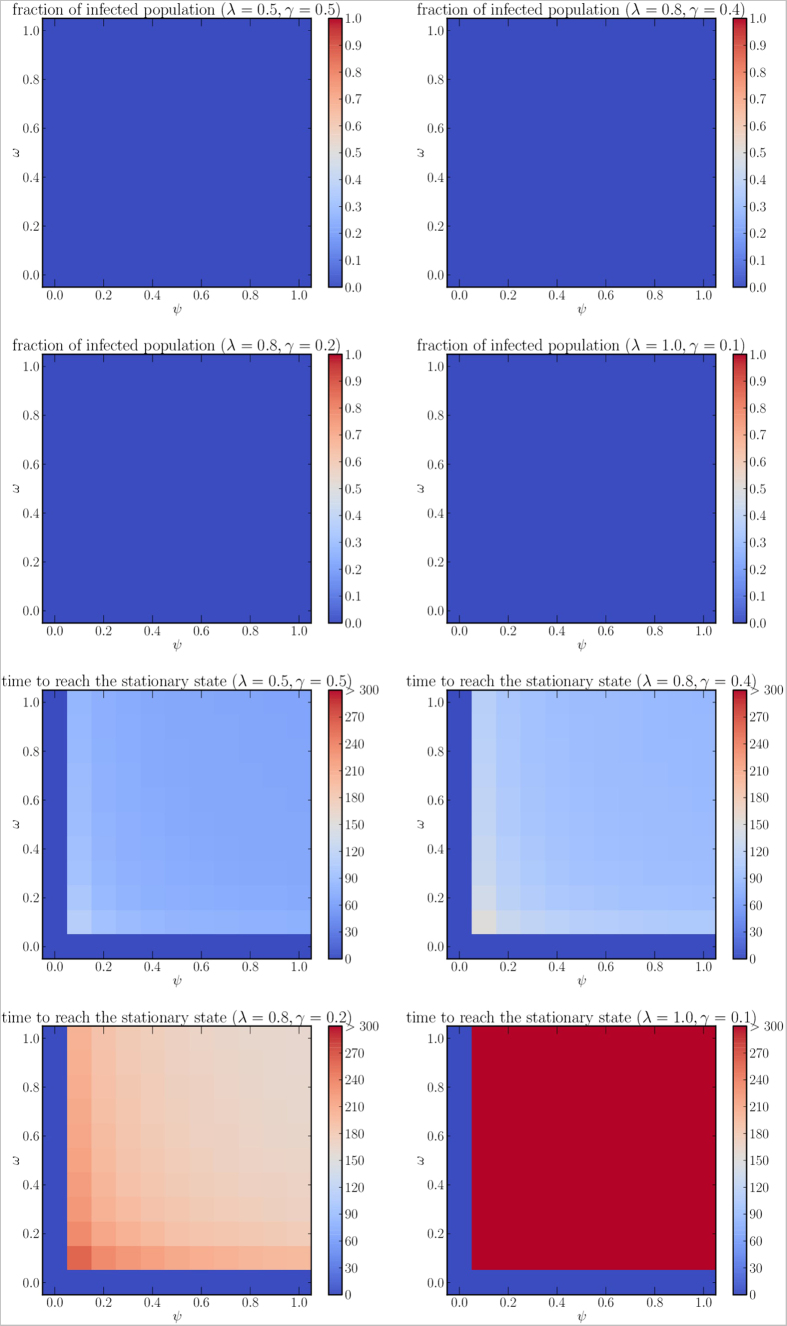
Fraction of infected population at the stationary state (first two rows) and time required to reach the stationary state (last two rows), for different combinations of 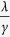 (1, 2, 4, 10, respectively) and *ξ* = 0.

**Figure 9:**
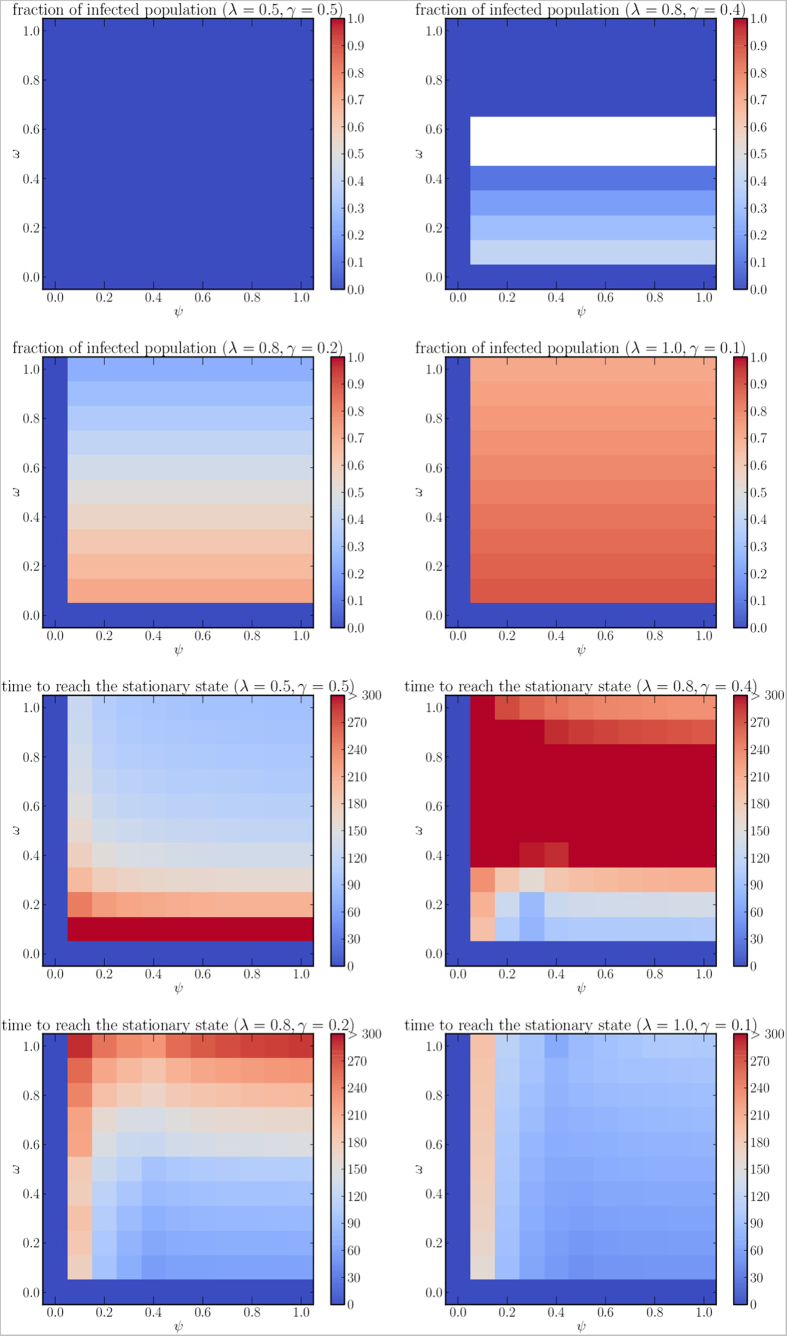
Fraction of infected population at the stationary state (first two rows) and time required to reach the stationary state (last two rows), for different combinations of 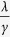 (1, 2, 4, 10, respectively) and *ξ* = 0.5. White spaces show that no stationary state is reached during the period of observation.

In Figs. 8,9 we show the density plots for *ω* and *ψ* when *ξ* is constant. In particular, we analyze the scenario for *ξ* = 0 (Fig. 8), which might represent dissemination of information about vaccination campaigns (individuals who have been administered vaccination do not lose immunity). For every combination of parameters we have absence of endemic state even with the highest considered value of *r*_0_. The two parameters that represent how individuals are likely to get involved in the immunization and in the information spreading (*ω* and *ψ*, respectively) seem to have similar impact on the delay of the infection.

The value *ξ* = 0.5 (Fig. 9) might describe a scenario in which the information is about a good practice (e.g., boiling water, using mosquito nets, etc.), which loses its effectiveness or it is stopped being used by a person with rate *ξ*. For this case we can notice that the fraction of infected population is independent from *ψ*, as rows in the density plot are of the same color. This suggests that, for this scenario, the rate at which people lose immunity does not affect the final size of the endemic state.

## Discussion

In this paper we have presented a model that describes the spreading of disease in a population where individuals move between geographic areas. We have shown the evolution of the disease and we have evaluated two types of countermeasures, namely the quarantine of central geographic areas and a collaborative information campaign among the population. Both the mobility and the underlying social structure have been extracted from cellular network records. Simulations based on this data show that travel restrictions are ineffective to contain the global epidemic, as shown in previous studies^29^. Simulations also show that information campaigns might be effective countermeasures and they can be a viable alternative to regional quarantine. However, our work is bound with certain limitations primarily due to the nature of the available data.

First, in this study, locations are aggregated at the level of *sous-prfectures*, the third-level administrative units in Ivory Coast. While coarse (the average sous-prefecture area is around 820 *km*^2^), we believe that the location granularity is appropriate for the problem at hand. In fact, an administrative unit of the territory is supposed to reflect its economic, social, cultural aspects, all factors which influence people behavior when moving and communicating. More importantly, it defines a geographical area over which an administrative authority has jurisdiction, can take decisions and implement policies. Furthermore, while cellular data can be analyzed at finer granularity, this comes with considerable inaccuracy: contrary to public belief, localizing accurately every device in a cellular network is not trivial. In fact, a phone is often not connected to the closest antenna, since several network-related factors, such as load balancing, come into play. Thus, it is unlikely that access to finer-grain data warrants additional analysis.

Second, while SIS/SIR models are commonly associated with the spreading of diseases, they can be used to model spreading processes in general^30^, e.g., spreading of ideas, beliefs, opinions, and may not be the most realistic in each of these domains. Other approaches, such as threshold models^31^, have also been proposed to model collective behavior. Our work is the first to consider orthogonal, yet interacting, processes on a multiplex network for disease containment. Finding the most realistic models that describe information dissemination and disease propagation is not the goal of this study.

Finally, in general, the mobility and communication matrices can be time-varying, and they can be adjusted according to seasonal trends or real-time data at each step, for example following estimates based on historical data. However, in order to simplify the presentation, in this study we use matrices that do not change over time. It is worth remarking that our approach can be straightforwardly generalized to account for network descriptors and activity driven modeling of time-varying networks^32^,^33^,^34^,^35^,^36^,^37^,^38^.

## Methods

The data provided for the D4D Challenge^24^ consists of four datasets (identified by the labels SET1, SET2, SET3, SET4), containing information about user mobility and call patterns at various levels of granularity and time duration. Here we discuss how we used these datasets to build mobility and information spreading models, introduced in the *Results* section.

Two datasets contain information about mobility and communication patterns at macroscopic level. The SET3 dataset contains the trajectories of 50,000 randomly-selected individuals, at a sub-prefecture level resolution, for five months. This dataset can be used to estimate the probability that an individual moves from the sub-prefecture *i* to the sub-prefecture *j*:
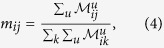
where 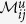 is the number of times user *u* moves from the sub-prefecture *i* to *j*. The numerator counts how many times users who are in *i* move to *j*; the denominator normalizes this number by the total number of transitions from *i* to any sub-prefecture *k*. When *i* = *j*, *m*_*ij*_ is defined as the number of people who remain within their own metapopulation. Using these values we build a mobility matrix *M*. The matrix is not built on any specific time window – its time granularity is the time granularity that is inherent to the dataset. By using this matrix, we model human mobility in the country as a Markov process^39^. We observe that the matrix is quite sparse and the highest values are concentrated along the diagonal. As the representation is in logarithmic scale, this demonstrates that the movement between sub-prefectures is present, but rather uncommon. This matrix represents the mobility layer of the multiplex network used in our model. In addition, we use information found in SET3 to allocate the population to different geographic areas. The number of people in each metapopulation in general is not constant.

The SET1 dataset contains the number and the duration of calls between pairs of cell phone towers, aggregated by hour. This dataset provides macroscopic information about communication in the country. We associate cell phone towers with the sub-prefecture they are located in, by using the supplied geographic position. Then, we evaluate the probability of a call being established between sub-prefectures *i* and *j* with:
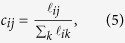
where 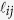 is the number of phone calls initiated from the sub-prefecture *i* and directed to the sub-prefecture *j*, during the entire period of observation. The term at denominator indicates the total communication flux out of *i* and it is used to normalize the probability. Using these values we build a calls matrix *C*. This matrix also shows high values along the diagonal, but it is distinctly denser, showing that calls between sub-prefectures are more common than movement. The vertical line at *x* = 60 identifies calls directed to the sub-prefecture that contains the capital. This matrix represents the communication layer of the multiplex network used in our model.

The other two datasets provide microscopic information about mobility and communication patterns between individuals. Although we do not use them for the analysis in this paper, we now briefly outline how they could be used. The SET2 dataset contains fine-grained individual trajectories of 50,000 randomly sampled individuals over two-week periods. This dataset could be used to estimate the number of potential connections that an individual might have in a certain area, served by a cell phone tower. The SET4 dataset contains time-varying ego-networks of 5,000 users, describing the network of communication in time-slots of 2 weeks. If two users are connected by a link in a time-slot, it means that *at least* one call occurred during the two weeks under consideration.

## Additional Information

**How to cite this article**: Lima, A. *et al.* Disease Containment Strategies based on Mobility and Information Dissemination. *Sci. Rep.* **5**, 10650; doi: 10.1038/srep10650 (2015).
